# Doping Carbon Nanotube Ethylene-Vinyl Acetate Thin Films for Touch-Sensitive Applications

**DOI:** 10.1021/acsaelm.4c02246

**Published:** 2025-05-29

**Authors:** Bernd K. Sturdza, Nicole Jacobus, Andre Bennett, Joshua Form, Louis Wood, M. Greyson Christoforo, Moritz K. Riede, Robin J. Nicholas

**Affiliations:** Department of Physics, Clarendon Laboratory, 6396 University of Oxford, Parks Road, Oxford OX1 3PU, U.K.

**Keywords:** transparent conductive films, carbon nanotubes, polymer wrapping, ethylene-vinyl acetate, adsorption p-doping, percolation, carbon nanotube-polymer films

## Abstract

Transparent conductive films are key components of many optoelectronic devices but are often made from either scarce or brittle materials like indium tin oxide. Carbon nanotube-polymer films offer an abundant and flexible alternative. Here, we report how the dimensions of the carbon nanotube raw material affect their thin film performance and thickness yield when processed with the polymer ethylene-vinyl acetate. We perform chemical doping with several halogenated metals and find the electron affinity of the metal to be a good indicator of p-doping effectiveness. We identify CuCl_2_ as low-cost alternative to the established gold chloride dopants. Optimising the dopant deposition method allows us to reduce the effect of doping on the optical transmittance. Percolation analysis of our films demonstrates that optimized single-walled carbon nanotube-ethylene-vinyl acetate films show no sign of percolation effects down to thicknesses of 5 nm. Finally, we produce transparent touch-sensitive devices. Comparing several of these devices, we find a linear relationship between the sheet resistance and the on/off ratio of the touch sensing that can be used to determine a threshold film thickness. Using doped carbon nanotube-ethylene-vinyl acetate films increases the on/off ratio and allows us to fabricate touch-sensitive devices with an on/off ratio of 10 at 95% optical transmittance. This clearly demonstrates the potential of these films for transparent touch-sensitive applications.

## Graphical abstract

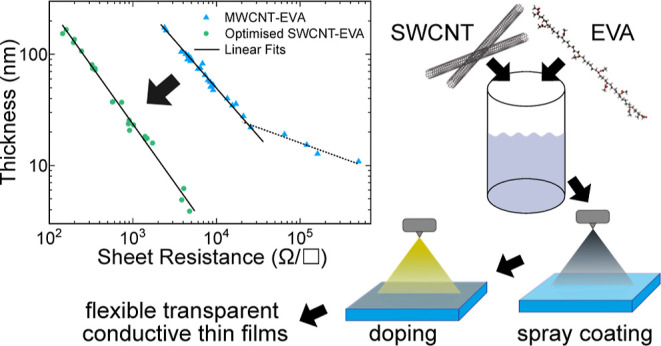

## Introduction

A future of smart machines and interconnected devices with an Internet of things will be accompanied by an increasing demand for electronics with displays, sensors, batteries or small photovoltaic cells. Transparent conductive films (TCFs) are a fundamental part in many of these, allowing the coupling of light into or out of an electronic device.

The two parameters of interest when comparing TCFs are the sheet resistance *R*_*s*_ and the transmittance *T* which is typically evaluated at 550 nm, where the luminosity curve of the human eye has its maximum.^?^ In order to relate the optical and electrical properties of carbon nanotube (CNT) films, we express *T* in terms of the film thickness *t* as^1^

1 $$T={\left(1+\frac{Z_{0}}{2}\sigma_{op}t\right)}^{-2}$$

with the vacuum impedance *Z*_0_ = 1/ϵ_0_ *c* = 377 Ω and the optical conductivity σ_op_ which has been shown to be equal to σ_op_ = 1.7 × 10^4^ S/m for CNT films.^2,3^ From this we can find a relation between *T* and *R*_*s*_

2 $$T={\left(1+\frac{Z_{0}\sigma_{op}}{2R_{s}\sigma_{dc}}\right)}^{-2}$$

where σ_dc_ is the dc conductivity of the CNT film.^4^ The ratio σ_dc_/σ_op_ serves as a figure of merit (FoM) for comparison of TCF performance, where large FoM values correspond to low *R*_*s*_ and high *T*.

Traditionally, metal oxides like indium tin oxide (ITO) perform really well as TCF materials and are thus predominantly used in optoelectronic applications.^5,6^ However, their crystalline structure limits their performance in flexible and stretchable applications.^7–9^ In addition, the scarcity of materials such as indium is becoming a limiting factor in the deployment of new technologies, i.e. current estimates suggest only 25% of global solar cell demand for indium can be met, posing a significant challenge for the energy transition.^?^ It is therefore crucial to replace indium where possible, e.g. in applications with low performance requirements such as touch sensors,^10^ so sufficient supply is available for applications where it cannot be replaced yet.

In recent years, materials such as graphene, silver nanowires, conducting polymers, and metal mesh have been investigated as potential TCF replacements.^7,11–14^ However, like ITO, many of these materials possess poor cost-performance ratios and struggle to satisfy the requirements of modern optoelectronic devices.^15,16^

By contrast, CNTs possess a set of optical, electronic, and mechanical characteristics that make them a promising alternative for TCF applications.^13,17^ CNTs benefit particularly from the ability to produce more stable flexible films with higher mechanical strength than competitor materials.^18^

Recent advances in synthesis have demonstrated that CNTs can not only be made from traditional carbon sources, like ethylene, but also from plastic waste^19^ or directly from CO_2_.^20^

However, the remaining key challenge is to find a low-cost and scalable processing route.

While high-performance CNT films are typically prepared via the dry transfer method, for large-scale applications solution processing is required instead.^13,21^ An established method to solubilize CNTs is polymer wrapping. This noncovalent functionalization prevents agglomeration and allows the processing of individualized CNTs.

We have previously established that the nonconjugated copolymer ethylene-vinyl acetate (EVA) allows the dispersion and deposition of individual CNTs to form CNT-EVA thin films.^22^ We have since significantly improved the processing and performance of CNT-EVA films.^23^ Here, we investigate the effects of CNT dimensions on the TCF performance of flexible CNT-EVA films. We compare various candidates for chemical doping and identify a potential performance indicator of metal halogen-based dopants. We study percolation effects in our films at application relevant thicknesses and find large differences between multi- and single-walled CNTS. Finally, we put these advances into practice by making capacitive touch-sensitive devices from our CNT-EVA films that go well beyond previously established CNT touch-sense devices.^24^

## Results and Discussion

### Impact of CNT Diameter and Length

Carbon nanotubes have become commercially available in a wide range of diameters, lengths, purities, and prices in both single- and multiwalled structures. In general, multiwalled carbon nanotubes (MWCNTs) cost less than single-walled carbon nanotubes (SWCNTs) and are expected to perform worse in TCF applications due to the larger amount of carbon and consequently absorption of light per unit length of nanotube. However, MWCNTs do not possess an electronic band gap and thus do not suffer from high intertube resistances due to Schottky barriers. To identify which of these two effects dominates in our system, we compare both multiple MWCNT and SWCNT raw materials, listed in Table S1.

Further CNT raw materials were investigated, but did not provide sufficient yield in the preparation process to produce relevant CNT-EVA thin films. For all of the CNT types listed, multiple batches (*n* ≥ 10) of CNT-EVA solution were prepared following the description by Mazzotta et al.^22^ and thin films were deposited. The processing parameters were varied between batches of the same CNT material to find ideal processing conditions. The same range of processing parameters were used for all CNT types.

The two metrics considered for the resulting films (Figure 1) are the TCF figure of merit σ_dc_/σ_op_ and the film thickness achieved, allowing us to quantify the processing yield of different CNTs with EVA.

**Figure 1:**
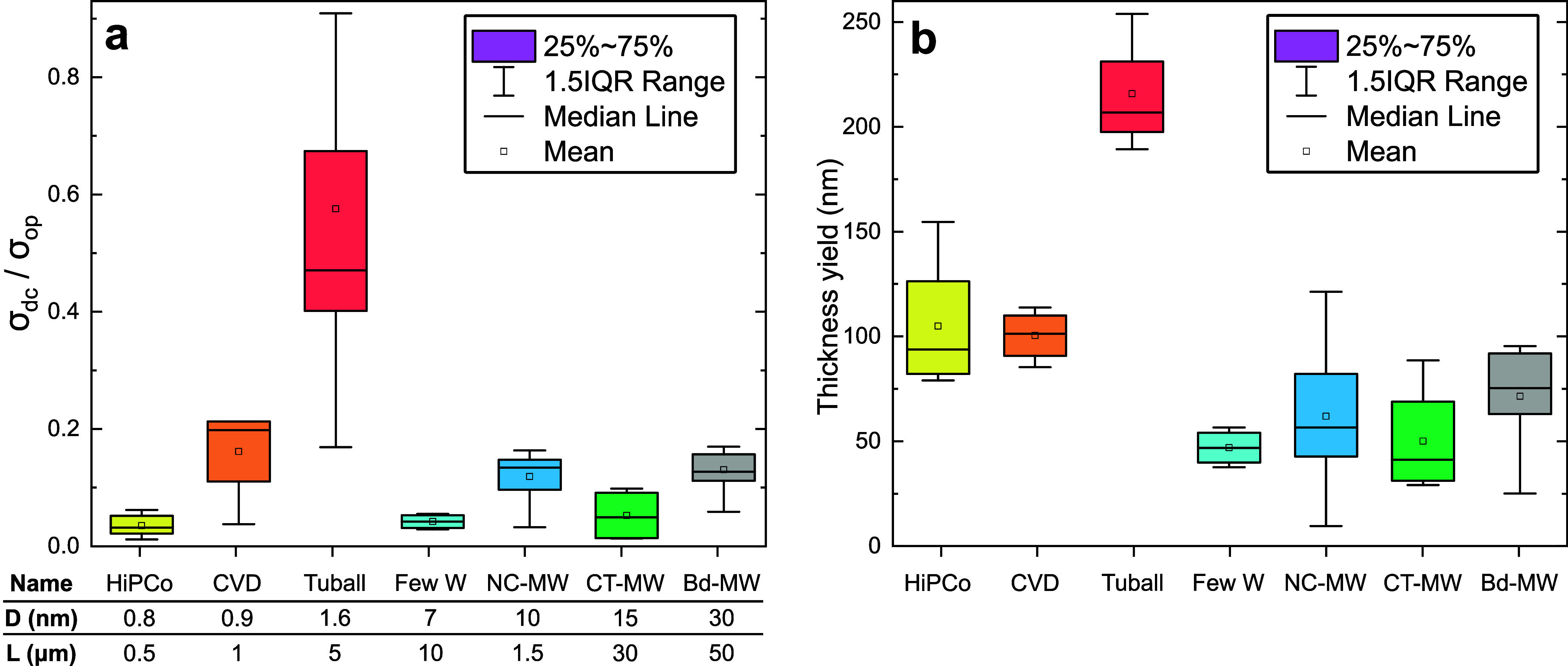
Thin film performance for different CNT types. The labels on the *x*-axis of the box plots indicate the different CNT types with nanotube diameter *D* and length *L*. Detailed CNT properties are given in Table S1. (a) TCF figure of merit for the different CNTs. (b) Thin film thickness yield with standardized processing for the different CNT types.

The thickness yield given in Figure 1b was derived from the transmittance at 550 nm via eq 1. This was confirmed for a number of films with Dektak measurements of the film thickness. We find large differences in this yield, which can be attributed to the dimensions of the CNTs. All single-walled CNTs give higher yields than any of the multiwalled CNTs. The yield for the larger diameter Tuball SWCNTs, which is estimated to be 90% absolute, is more than twice as high as for the sub-1 nm SWCNTs.

The nanotube length seems to be the main factor determining the performance of SWCNT films. HiPCo and CVD tubes have nearly identical diameters, but the longer CVD tubes show significantly higher FoM. For the even longer Tuball SWCNTs, the FoM is further enhanced. Furthermore, we find that EVA does not wrap tubes with diameters above 30 nm well and for CNTs longer than 50 μm dispersion treatments and the separation of wrapped tubes from bundles via centrifugation fails. Since both FoM and thickness yield are negligible for these CNTs the data are not shown. Tuball SWCNTs perform best in both FoM and thickness yield. The yield is in fact so high that at sufficient CNT concentrations the purification steps of the processing are impaired, causing the large spread of σ_dc_/σ_op_ in these films. At reduced CNT concentrations, the performance of Tuball SWCNTs improved significantly and we consequently chose these large-diameter SWCNTs for the following experiments.

### Chemical Doping

Doping is a standard technique to increase the charge carrier density in semiconductors. In contrast to inorganic semiconductors, where this is typically realized by lattice doping, dopant adsorption has proven to be more effective in CNTs.^10^ This process describes charge transfer between dopant molecules and CNTs, the resulting Coulomb attraction between positive and negative charges stabilizes the dopant on the CNT sidewall.^25,26^ In CNT networks doping has the beneficial effect of removing Schottky barriers between semiconducting and metallic tubes by making more of the semiconducting tubes metallic and thereby reducing effective intertube junction resistances. Due to their low electron affinity, CNTs are typically p-doped. Various dopants have been identified, the most promising being acids and halogenated metals.^27,28^ Nitric acid (HNO_3_) has been widely used and has been shown to improve the electrical conductivity of CNT films by a factor of 10,^29^ chloroauric acid (HAuCl_4_) by factors of up to 13.5.^30^ Zhou et al. have used copper halides to achieve improvements up to a factor of 10 as well and argue that these dopants have the additional benefit of adding interconnections between CNTs in the film, further lowering the sheet resistance.^31^

In our experiments, we chose a range of different halogenated metals and two acids and applied them to Tuball SWCNT-EVA films via 3× horizontal spray deposition (see Figure S2). Nitric acid proved incompatible with CNT-EVA films, as it decomposed EVA and detached thin films from the substrate. The remaining halogenated metals were successfully used as dopants of SWCNT-EVA films, results are given as box plots in Figure 2 with identical settings to the box plots in Figure 1.

**Figure 2:**
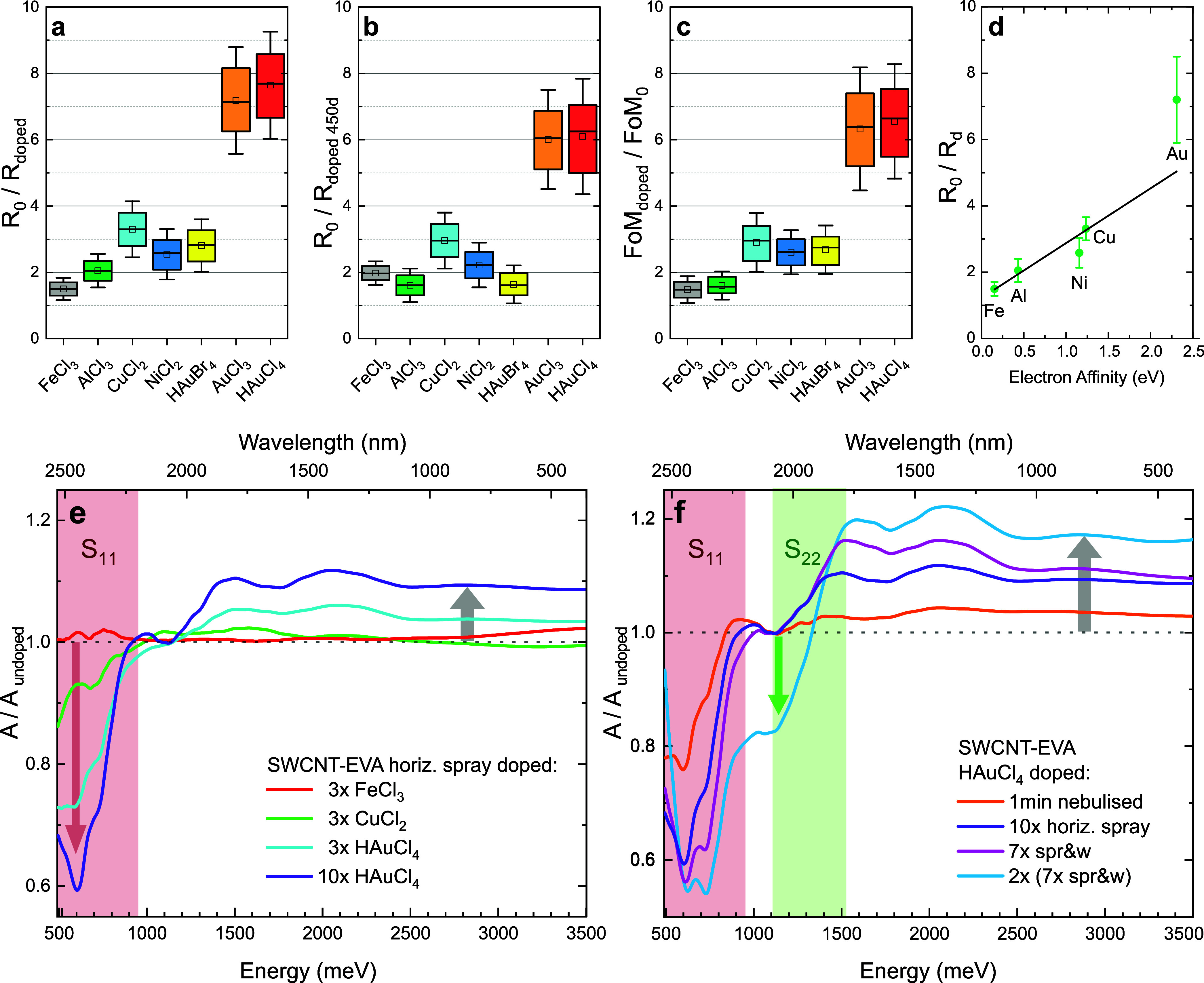
Dopants and their effect on SWCNT-EVA films. (a–c) Relative change in sheet resistance, sheet resistance after 450 days ambient storage and figure of merit upon doping. (d) Conductivity improvement of SWCNT-EVA films doped with different chlorinated metals plotted against the metals’ electron affinity. A linear fit is shown. (e,f) Relative change in absorbance upon chemical doping with different dopants and deposition techniques. The first (S_11_) and second (S_22_) semiconducting interband transitions are emphasized in red and green.

All halogenated metals improve the conductivity of our SWCNT-EVA films, see Figure 2a. However, the two gold chloride-based dopants outperform the other dopants clearly, with HAuCl_4_ providing the most promising results, reducing the sheet resistance of our films by factors of 7.6 ± 1.

To investigate the long-term stability of doped films, an essential criterion for applications, the measurements were repeated after ambient storage at 20 °C for 450 days, see Figure 2b. The films did not show any visible degradation after this time. While the conductivity of FeCl_3_ and CuCl_2_ doped films improved slightly over time, for AlCl_3_, NiCl_2_ and HAuBr_4_ doped films the conductivity showed a small decrease. Most adversely affected by aging were the two gold chloride-based dopants, with a 20% decrease in conductivity.

For a perfectly transparent dopant, the change in electrical conductivity would be equal to the change in the TCF figure of merit. However, we found that all dopants either absorb light in the visible range or react into compounds that do in ambient conditions, which means the increase in electrical conductivity is accompanied by a decrease in transmittance. To reflect both of these changes, we compare the TCF FoM σ_dc_/σ_op_ in Figure 2c. While the FoM improvements are only about 10% less than the conductivity improvements in Figure 2a, this still suggests that the amount of dopant applied needs to be chosen carefully to limit the reduction of transmittance.

The reason why gold chloride-based dopants outperform the other halogenated metals can be found in the underlying chemical reactions of the doping process.^25^ These are based on the transfer of electrons from the CNTs to the dopant molecules. To prevent the strongly electronegative halogen from drawing electrons from the metal instead of the CNTs upon disproportionation of the dopant, large electron affinities are required for the metal as well.

For the chlorinated metals, the metal electron affinity is plotted against the conductivity improvement in Figure 2d. The dopant performance increases with the electron affinity of the dopant metal. A linear fit is shown, but the data set is insufficient to distinguish a linear from a higher-order dependence. We hypothesize that the low performance of HAuBr_4_ compared to HAuCl_4_ might be linked to the lower electron affinity of Bromine compared to Chlorine.

To further investigate the doping mechanism, we performed absorbance measurements in the range of 500–3500 meV (350–2500 nm) of doped SWCNT-EVA films for different dopants, plotted in Figure 2e relative to the absorbance of the pristine films. As expected, we find that the absorbance of doped films is slightly increased near the peak sensitivity of the human eye at 2.25 eV (550 nm), which is the wavelength used for calculating FoM.

The energy range of the first interband transition (S_11_) of the semiconducting SWCNTs is shaded in red. Chemical p-doping of CNTs shifts the Fermi level down into the first valence band and consequently quenches the absorption of the first interband transition because there are fewer occupied states in the valence band. We find that this effect correlates with the increase in the electrical conductivity of our films. For FeCl_3_, achieving conductivity improvements of 150%, there is no appreciable change in the S_11_ absorption, whereas for CuCl_2_ (conductivity improvement of 330%) the S_11_ absorption is reduced by 5–10%. For HAuCl_4_ (conductivity improvement of 760%) we observe a 30% reduction in S_11_ absorption. This suggests that FeCl_3_ does not induce transfer of electrons from CNTs, but CuCl_2_ and HAuCl_4_ do, which agrees with our previous hypothesis about the importance of the electron affinity of the dopant metal. We conclude that the positive effect of FeCl_3_ on the electrical conductivity of our SWCNT-EVA films is caused by adding interconnections between CNTs in the film rather than chemical p-doping.^31^

The purple graph in Figure 2e represents a 10× HAuCl_4_ spray-doped sample. Compared to the 3× spray-doped film, the effect on S_11_ intensifies from a 30% to a 40% reduction in absorbance, but the absorption in the visible region also increases substantially. If even more HAuCl_4_ solution is applied (blue graph in Figure 2f), we find that the second interband transition S_22_ (green area) is partially quenched as well, suggesting that sufficient electrons have been removed from the CNTs to push the Fermi level down into the second valence band. This allows us to estimate the concentration of induced electron holes in the CNTs to *p* = 0.20 ± 0.05 nm^–1^ ^32,33^ suggesting there is an induced electron hole for every 5 nm of SWCNT. This result demonstrates that SWCNT-EVA films can be heavily p-doped despite the presence of the insulating EVA polymer in the films.

However, high doping concentrations are desirable only if a high transmittance can be maintained. On account of this, we compared four different deposition methods (see Figure S2), for the best performing dopant HAuCl_4_. We found that the deposition method plays a key role in dopant-induced absorbance in the visible region, see Figure 2f. This is connected to the formation of dopant clusters as the solvent evaporates. These clusters are detrimental to performance, as they do not contribute to electrical conduction but absorb light. For simple drop-casting of the dopant (not shown), there is a visible coffee ring effect upon solvent evaporation. For the dopant solution applied with a spray nozzle, the droplet size is still too large to prevent the formation of dopant clusters.

Therefore, we adapt the nebulizer doping technique reported by Tsapenko et al.^30^ to minimize dopant droplet size and additionally heated our substrates to 50 °C during dopant deposition. This accelerates solvent evaporation and reduces optical absorption of the dopant layer at 550 nm from 8% for simple drop-casting to 2% for the optimized nebulizer technique at identical dopant concentrations.

### Percolation Effects

CNT thin films can be interpreted as networks of randomly orientated rods. Upon lowering the network density, areas of the network become isolated and stop contributing to charge transport. This effect is called percolation and is a common issue among nanostructured transparent conductors. If the film thickness falls below a certain value called *t*_min_, transport properties start to decay from the bulk behavior, and the dc conductivity decreases with decreasing thickness.

We compare the transmission and sheet resistance of previously established Nanocyl MWCNT-EVA films^22^ with Tuball SWCNT-EVA films, optimized as previously described^23^ and doped, in Figure 3a.

**Figure 3:**
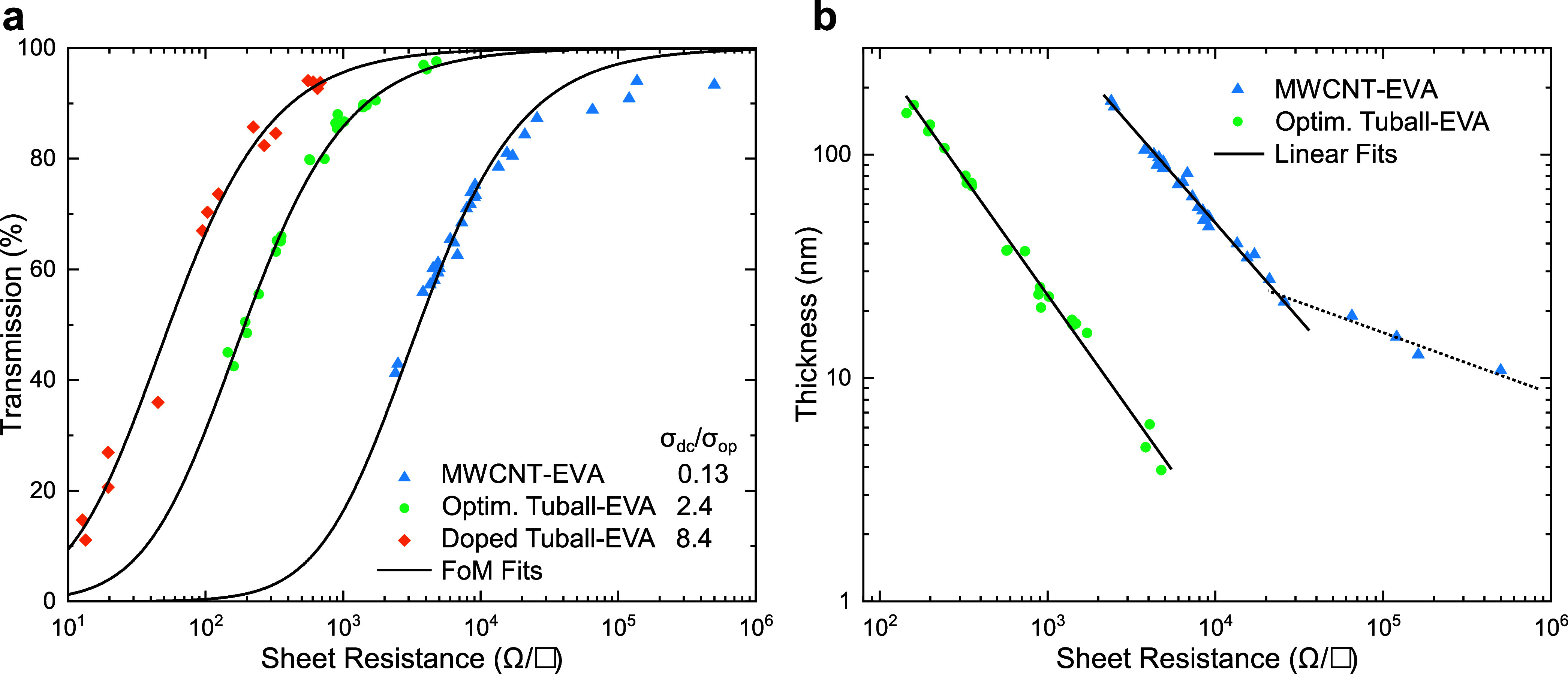
Percolation analysis of CNT-EVA thin films. (a) Transmission and sheet resistance for previously established Nanocyl MWCNT-EVA and optimized Tuball SWCNT-EVA films shown together with bulk model FoM fits. (b) Log–log plot of the same data with the transmission converted to film thickness via eq 1. While all optimized films follow the bulk model, MWCNT films are affected by percolation at thicknesses below 20 nm. The percolation regime is indicated by the dotted line.

The black lines are fits to the data after eq 2 following the bulk conduction model. Apart from the obvious difference in TCF performance, the fits highlight the deviation from bulk behavior at high transmission for MWCNT-EVA films. This trend becomes more obvious in Figure 3b, where the film thickness calculated using eq 1 is plotted against the sheet resistance. While for the optimized Tuball-EVA films all samples follow the same linear trend indicating bulk-like behavior, there are two linear regimes present for MWCNT-EVA data. At film thicknesses below *t*_min_ = 20 ± 4 nm the sheet resistance starts to increase much faster with decreasing thickness due to percolation effects. *t*_min_ has been linked to the smallest dimension *D* of the nanostructure via *t*_min_ = 2.33*D*.^34^ For Nanocyl MWCNTs with a diameter of *D* = 9.5 nm this predicts *t*_min_ = 22 nm which is in good agreement with our data.

In contrast, the smaller diameter Tuball tubes (*D* = 1.6 nm) are expected to have a much smaller *t*_min_ = 3.7 nm and our films show no sign of percolation effects down to thicknesses of 5 nm, making them suitable for high-transmittance applications with up to *T* = 95%. Furthermore, this confirms our observation from SEM images (Figure S1) that the optimized CNT-EVA films consist of individually wrapped tubes rather than bundles, since bundles would fall into the percolation regime at these low film thicknesses.

### SWCNT-EVA as Transparent Touch-Sensitive Thin Films

To demonstrate the high performance of our optimized films, we designed flexible touch-sensitive SWCNT-EVA thin film devices. This can be done with a SparkFun Electronics Teensy 3.2 K20 microcontroller that contains a touch-sense function measuring the ground-coupled capacitance connected to a pin. Upon touching the CNT film, its capacitance is increased, and the touch-sense function returns a larger output value. For a proof-of-concept device, we sprayed a 4 × 4 pixel matrix of SWCNT-EVA film on a flexible 10 × 10 cm^2^ PET substrate (Figure 4a) and connected the individual rows and columns to the microcontroller with conductive carbon tape contacts.

**Figure 4:**
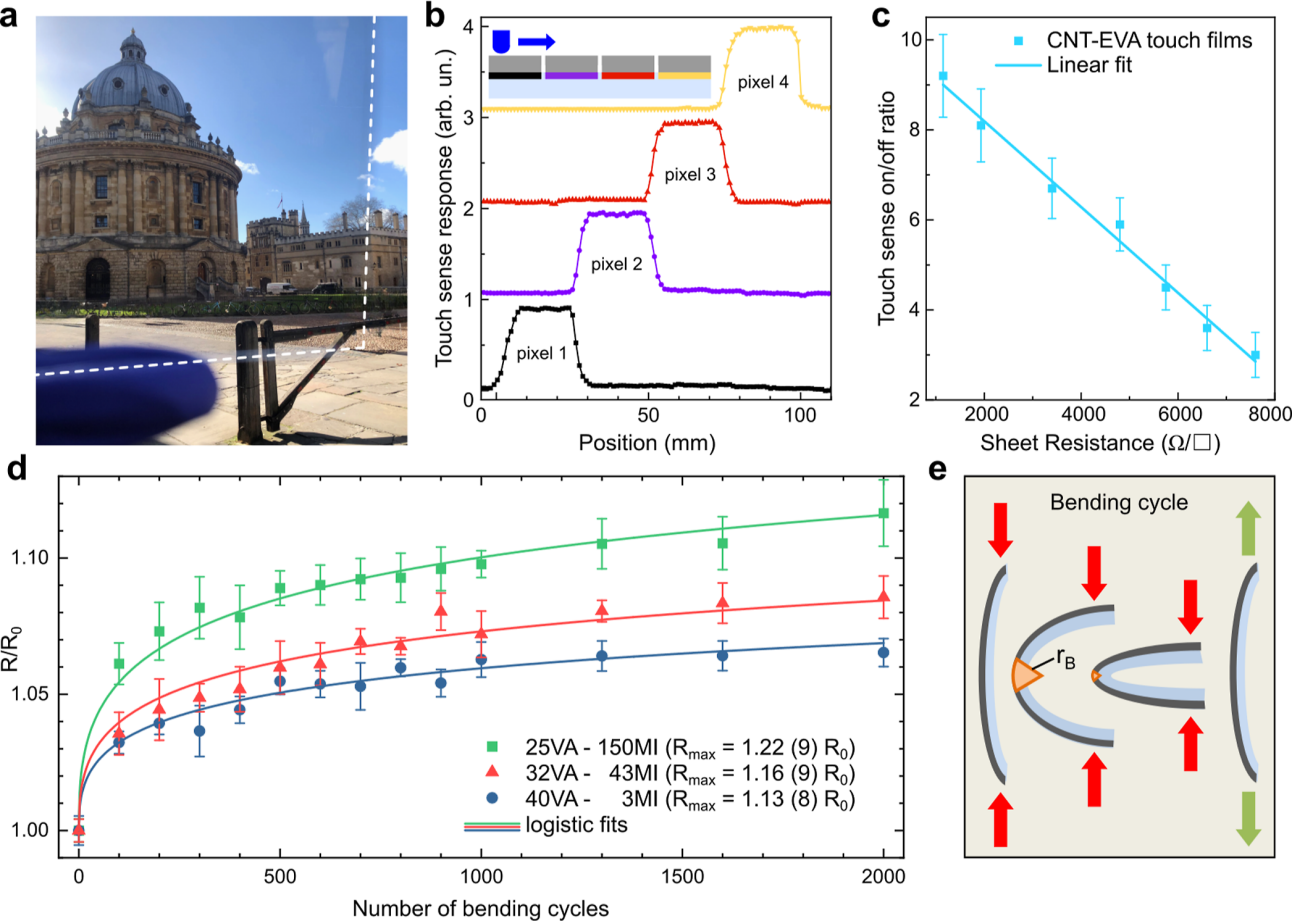
SWCNT-EVA touch-sense devices. (a) Photograph taken through a CNT-EVA film on PET, demonstrating the preserved high optical clarity. (b) Touch sense response of a line of four pixels when touched with a narrow object. The inset schematically depicts the experiment. (c) The on/off ratio of SWCNT-EVA touch-sense films with different doping concentrations as a function of the sheet resistance together with a linear fit. (d) Increase of electrical resistance (*R*) of CNT-EVA films at the line of highest strain for repeated bending for three different EVA polymers. Logistic fits provide the asymptotic maximum resistance for infinite bending cycles. (e) Schematic of a bending cylce with bending radius (*r*_B_ = 5 mm) and the line of highest strain shown in orange.

The pixel layout replicates the design of standard touchscreen devices and allows us to identify where the film was touched. The touch sense response for moving a narrow object across a four-pixel line is shown in Figure 4b. The devices performed well and showed no signs of degradation after being touched with gloves for extended periods of time. In fact, the sensitivity of the device was sufficient to detect the change in capacitance produced by the proximity of the finger several centimeters away. This allows CNT-EVA touch-sense films to be used without being physically touched, critical for applications such as touch-free sensors.

The upper limit for the sheet resistance of touch-sensitive transparent conductors is typically given as 1 kΩ/□^16^ which is just above of what our undoped SWCNT-EVA films achieve at 85% transmittance. We therefore investigated whether doped films could work as touch-sensitive devices at a higher transmittance. For this purpose, we fabricated several touch-sensitive devices by exposing 97% transmittant CNT-EVA films to varying amounts of HAuCl_4_ dopant. The doping incrementally reduced the sheet resistance from initially 7.6 kΩ/□ to 1.2 kΩ/□ for the film with the most dopant exposure. We found that the touch sense on/off ratio, defined as the ratio between the voltage signal amplitude when a pixel was touched and the baseline amplitude, correlates linearly with the sheet resistance of the SWCNT-EVA thin film in the probed range (Figure 4c). This shows that doped CNT-EVA films can produce the same touch sense on/off ratios (≈10) with much higher transmittances than undoped films (95% vs 85%). Furthermore, it is worth noting that even the undoped CNT-EVA film with 7.6 kΩ/□ had a touch sense on/off ratio of 3 at a transmittance of 97% and a thickness of less than 10 nm, which is still higher than on/off ratios of previously reported CNT-based touch-sense devices.^24^ This demonstrates that undoped SWCNT-EVA thin films can produce functional touch-sense films at extremely low thicknesses and transmittance values well above 85%.

The durability of CNT-EVA touch-sensitive films under bending strain is a critical parameter for flexible applications, e.g. in wearables. We have previously established that the properties of the copolymer EVA can be chosen to tune the mechanical properties of CNT-EVA films in accordance with specific applications.^23^ Since the two monomers, ethylene and vinyl acetate, possess the same size in direction of the polymer chain, it is expected that the monomer ratio does not affect the polymer’s ability to wrap CNTs. This matches our observations.

Here, we expose CNT-EVA films made with three different types of EVA polymer to 2000 bending cycles and measure the change in sheet resistance at the line of highest strain (Figure 4d). This will provide an upper limit for the loss of sensitivity in a flexible touch device.

The three EVA polymers investigated differ by their vinyl acetate wt % content (VA) and their melt index (MI), which is an industry standard for characterizing polymer chain length and is inversely related to molecular weight. Generally, a higher VA content reduces crystallinity and thus tensile strength and stiffness modulus but improves stress crack resistance.^35,36^ Similarly, longer chains, or lower MI values, improve mechanical properties at the cost of solubility.^35^ Therefore, we expect EVA polymers with higher VA content and lower MI values to perform better in flexible applications.

In our experiments, the sheet resistance of all three samples increases by about 5% during the first 100 bending cycles. During a bending cycle (Figure 4e), a force is applied to opposite edges of the CNT film until a predefined bending radius (*r*_B_ = 5 mm) is reached, whereupon the force is relieved and the film returns to a flat position.

Following the initial increase, the resistance of the films changes much more slowly in the following bending cycles, and can be described well by a logistic function. Global fitting of logistic functions (Figure S3) provides an asymptotic maximum for the resistance (*R*_max_) of the three samples after a large number of bending cycles.

The values of *R*_max_ are merely 13, 16, and 22% above the initial film resistance with the CNT-EVA film with the lowest VA content and highest melt index performing best, as expected.

## Conclusions

The comparison of several single- and multiwalled CNTs processed with EVA has shown that Tuball SWCNTs produce the best performing CNT-EVA transparent conductive films in our set while at the same time providing the highest processing yield. Our results confirm previous findings suggesting that CNT networks with individual SWCNTs of a large diameter, long length, high crystallinity, and heavy doping are the best candidates for transparent conductive films.^10^

We further demonstrated the chemical p-doping of SWCNT-EVA films for a range of halogenated metals. A large electron affinity of the metal appears to be a critical factor for successful p-doping, which we confirmed both in electrical and optical measurements. While the best-performing dopant was the established HAuCl_4_, we could establish CuCl_2_ as a promising low-cost alternative to the roughly 100 times more expensive gold-based dopants. Finally, we found the deposition method of the dopant solution to be critical to reducing the effect of doping on the optical transmittance which is linked to the formation of dopant clusters upon solvent evaporation.

Analyzing the percolation behavior of our films, we found that while MWCNT-EVA films fall into the percolative regime below film thicknesses of 20 nm, our optimized SWCNT-EVA films show no sign of percolation effects down to thicknesses of 5 nm.

Finally, we demonstrated that their improved TCF performance makes SWCNT-EVA films suitable for applications in transparent and flexible touch-sensitive devices. By making several of these touch-sensitive devices, we have found a linear relationship between the sheet resistance and the on/off ratio of the touch sensing that can be used to determine a threshold film thickness.

Using doped CNT-EVA films increases the on/off ratio and allows us to fabricate touch-sensitive devices with an on/off ratio of 10 at 95% optical transmittance. This demonstrates the large potential of CNT-EVA films for transparent touch-sensitive applications. Further research is needed to evaluate the performance of these films in full stack devices.

## Experimental Section

### CNT-EVA Solution Preparation

40 mg of EVA pellets (ELVAX, Dupont) with 25, 32, or 40 wt % vinyl acetate comonomer content (32 wt % was used unless otherwise noted) were dissolved in 100 mL chlorobenzene (Acros Organics, ACS reagent grade) by stirring at 50 °C overnight. 40 mg of Tuball SWCNTs (OCSiAl, 93% grade) were added to the solution and it was shear force mixed for 48 h at 10000 rpm while kept at a constant temperature of 28 °C. A custom shear force mixer was developed in partnership with Silverson Machines Ltd. for this project. It is based on the L5M-A-SU model with improved seals, a larger mixing head, and an integrated cooling system to prevent overheating. After shear force mixing, the mixture was centrifuged at 10 000*g* for 8 min and the supernatant collected. The precipitate containing impurities and nonfunctionalized CNTs was discarded. The supernatant was added to 200 mL of toluene and placed on a hot plate at 50 °C for 24 h to cause aggregation of nanotubes while keeping excess EVA polymer dissolved. After cooling back to room temperature, the mixture was centrifuged at 16 000*g* for 4 min or until the supernatant was fully transparent. The precipitate now contains pellets of functionalized CNTs, while excess EVA is removed with the supernatant. This excess polymer removal step is repeated once more and the CNT-EVA pellets are then dissolved in 500 mL of chloroform (Acros Organics, for HPCL grade, stabilized with 25 ppm amylene). This dispersion is stable for weeks and CNT aggregates can easily be broken up by a brief dispersion treatment prior spray deposition.

### Conductive Film Deposition

CNT-EVA films were spray coated with a custom-built open science hardware setup^38^ with the substrates held at 100 °C. PET or glass substrates were prepared as previously described.^22^

### Chemical Doping of CNT/EVA Films

Four different dopant deposition methods have been investigated (Figure S2). For every method, the corresponding dopant (Alfa Aesar, ACS grade 99%) was dissolved in ethanol (Merck, ACS grade) at a concentration of 5 mM. This solution was then deposited via either horizontal spray doping, vertical spray doping or nebulizer doping. The spray doping was done with a standard polyethylene terephthalate spray bottle, the nebulizing was done with an OMRON compressor nebulizer NE-C801 as previously described.^30^

### Transmittance Measurements

Transmittance data was acquired with a PerkinElmer LAMBDA 1050 UV–vis–NIR spectrophotometer using 1 nm steps.

### Sheet Resistance Measurements

4-point tungsten carbide probes from Jandel Engineering (0.634 mm spacing) and a Keithley 2450 source meter were used to measure sheet resistance. A sweep current from −0.2 to 0.2 mA was applied across the outer probes while the voltage across the inner probes was measured. The slope of the current–voltage data was first corrected with the factor π/ln(2) for the in-line configuration and then used to calculate the sheet resistance. For each sample, five spots across the film were measured and results averaged.

To prevent damaging the probes, doped films were measured with a van der Pauw geometry.^37^ For this purpose, small silver contacts were applied to the four corners of every sample with a paint brush and conductive silver ink. Current–voltage curves were taken as before but in two different directions. **Bending experiments** CNT-EVA films were subjected to 100 bending cycles with a bending radius of 5 mm, as depicted in Figure 4e. The sheet resistance was then measured at five spots along the line of highest strain in the in-line configuration as described above. This procedure was repeated until a total of 1000 cycles was reached at which point the measurement inverval was increased to 300 bending cycles.

### SEM Images

A Hitachi S-4300 SEM with an accelerating voltage of 10 kV was used to take SEM images.

## Supplementary Material



## Notes

Table of CNT properties, SEM data of CNT films, schematic of dopant deposition methods, global fit results of the change in sheet resistance of CNT-EVA films upon bending (PDF)
