# Supplementary material for: Doping Carbon Nanotube Ethylene-Vinyl Acetate Thin Films for Touch-Sensitive Applications
Source: ACS Appl Electron Mater. 2025 May 29;7(11):4738–46. doi: 10.1021/acsaelm.4c02246 (PMC12160056; doi:10.1021/acsaelm.4c02246)
Supplement: Supplementary file 1 [file el4c02246_si_001.pdf]

Supporting Information:

Doping carbon nanotube ethylene-vinyl acetate thin films for touch-sensitive applications

Bernd K. Sturdza,* Nicole Jacobus, Andre Bennett, Joshua Form, Louis Wood, M. Greyson Christoforo, Moritz K. Riede, and Robin J. Nicholas*

Department of Physics, Clarendon Laboratory, University of Oxford, Parks Road, Oxford OX1 3PU, UK

E-mail: bernd.sturdza@physics.ox.ac.uk; robin.nicholas@physics.ox.ac.uk

Table S1: **Properties of the CNT types compared in this work.** Values were adopted from the provided data sheets.[S1–S6]

CNT type	product name	Diameter (nm)	Length (μm)	Purity (wt%)	Price (€/g)
SWCNT	HiPco	0.85 ± 0.25	$0.1 - 1$	95	550
	CVD	0.9 ± 0.2	~ 1	95	1,300
	Tuball	1.6 ± 0.4	> 5	93	6
MWCNT	Nanocyl	~ 9.5	1.5	90	0.65
	Boron doped	~ 30	50	92	75
	Few walled	~ 7	> 10	92	150
	Cheaptubes 13-18	~ 15	~ 30	95	10

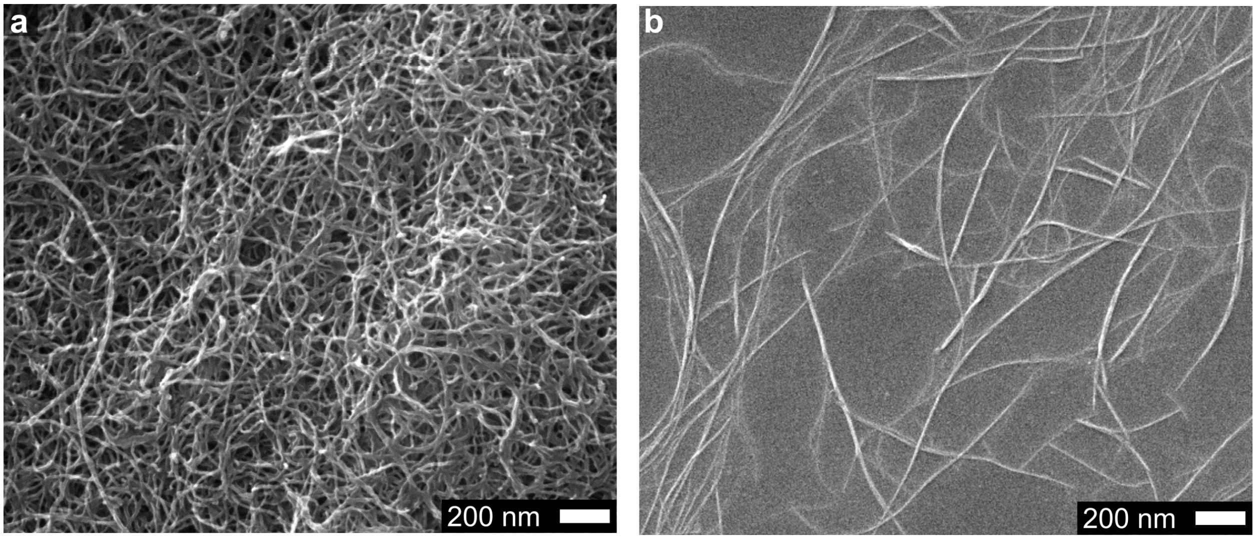

Figure S1: **SEM images of CNT-EVA on Silicon.** **a)** Nanocyl MWCNT-EVA film, **b)** dilute Tuball SWCNT-EVA film.

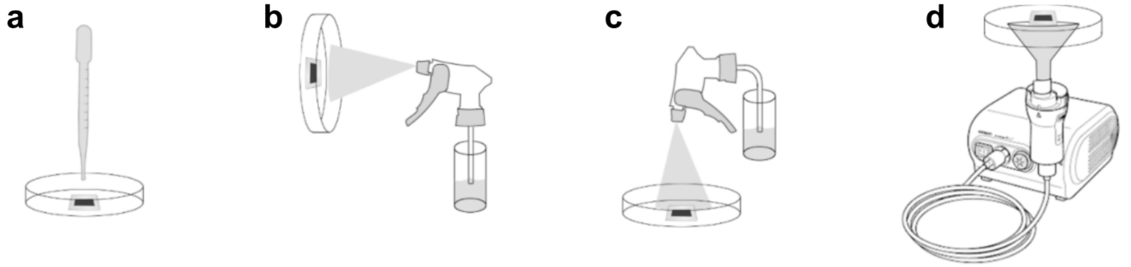

Figure S2: **Dopant deposition techniques.** The four different dopant deposition techniques investigated in this work are **a)** drop-casting, **b)** horizontal spraying, **c)** vertical spraying and **d)** nebulising with an OMRON compressor nebuliser NE-C801.

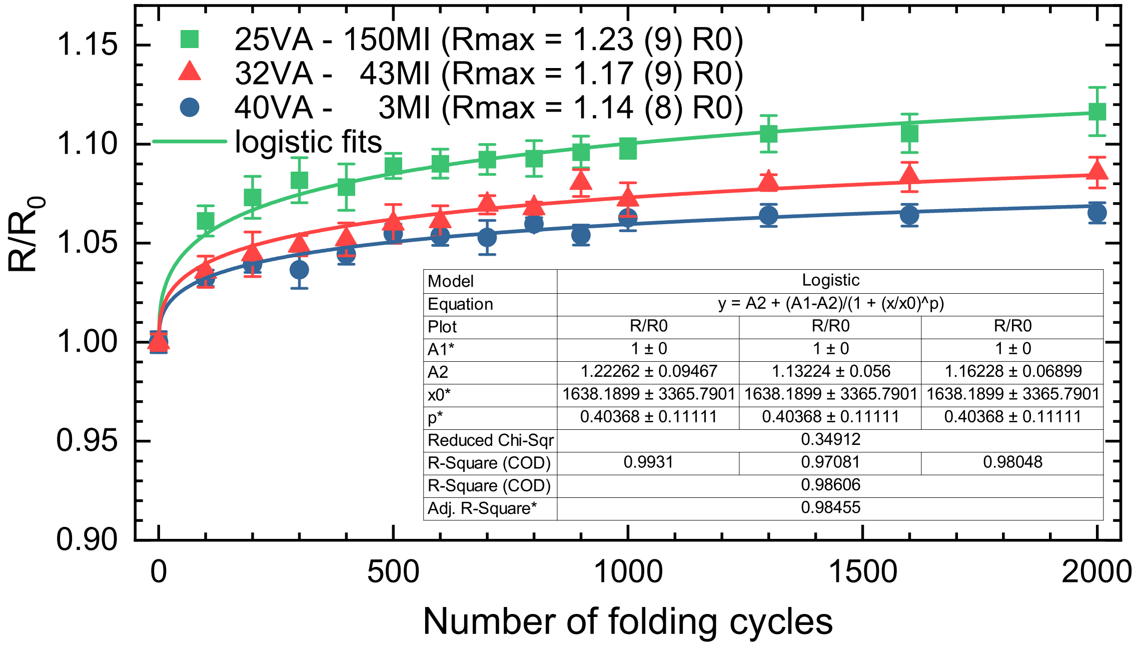

Figure S3: **Global fit of the resistance increase of a CNT-EVA touch-sense film upon bending with.**

References

(S1) NanoIntegris. HiPco® Single-Wall Carbon Nanotubes specifications sheet. https://nanointegris.com/our-products/hipco-small-diameter-swcnts/.

(S2) Sigma Aldrich CVD SWCNT 775533 specifications. https://www.sigmaaldrich.com/DE/de/product/aldrich/775533.

(S3) Ocsial Tuball R technical information. https://tuball.com.

(S4) Nanocyl Multi Wall Carbon Nanotubes NC7000 technical data sheet. www.nanocyl.com/product/nc7000/.

(S5) Cheaptubes Multi walled carbon nanotubes specifications. https://www.cheaptubes.com/product-category/multi-walled-carbon-nanotubes/.

(S6) Nanotechlabs Boron doped MWCNT Material Data Sheet. https://www.nanotechlabs.com/Nanotubes.html.
